# DNase I as a probe for unpolymerized actin: revisiting a classic tool for nuclear actin research

**DOI:** 10.1007/s00424-026-03158-z

**Published:** 2026-03-26

**Authors:** Anika Göpel, Laura Bauer, Dörthe M. Katschinski, Anke Zieseniss

**Affiliations:** Institute of Cardiovascular Physiology, University Medical Center Göttingen, Georg-August-University, Humboldtallee 23, 37073 Göttingen, Germany

**Keywords:** G-actin, F-actin, DNase I, Staining

## Abstract

Actin is a key component of the cytoskeleton and also plays diverse roles within the cell nucleus. While polymerized F-actin can be detected using a wide range of probes, reliable methods to identify unpolymerized (“G-”) actin in fixed cells are relatively limited. Fluorescently labeled DNase I has long served as a high-affinity probe for monomeric actin and has recently gained renewed interest in nuclear actin research. Here, we briefly review established methods for visualizing actin in the cytoplasm and nucleus and revisit the history and molecular basis of the DNase I–actin interaction that forms the basis for DNase I staining. We then highlight practical considerations for interpreting DNase I fluorescence signals, such as its binding to filament pointed ends, the influence of divalent cations on its enzymatic activity and DNA binding, and protocol-dependent variations. Notably, fixation conditions significantly influence staining outcomes, affecting the distribution of nuclear versus cytoplasmic signals. Overall, this review aims to provide a concise framework for effectively using DNase I staining to monitor cellular pools of unpolymerized actin in a consistent and interpretable way.

**Supplementary Information:**

The online version contains supplementary material available at 10.1007/s00424-026-03158-z.

## Introduction

As one of the most abundant proteins in eukaryotic cells, actin participates in a wide range of cytoplasmic and nuclear processes. In the cytoplasm, actin supports, among others, cell migration, shape changes, and intracellular transport. To fulfill these functions, actin undergoes precisely regulated cycles of polymerization and depolymerization, shifting between monomeric G-actin and filamentous F-actin [1]. F-actin forms a double-stranded, polar helix with two distinct ends: the barbed end (+) and the pointed end (-). Under polymerizing conditions, actin filaments elongate faster at the barbed end, whereas under depolymerizing conditions, monomer dissociation is also typically faster at the barbed end. In vivo, turnover rates vary greatly depending on the actin structure. For example, in fibroblast lamellipodia, basal actin polymerization and depolymerization remodel an appreciable fraction, i.e., ~ 3% of the filament network, on the order of seconds [2]. Various actin-binding proteins (ABPs) mediate these dynamics by capping, bundling, severing, or otherwise regulating filaments. Because the concentration of G-actin in the cytoplasm is well above the critical concentration required for spontaneous polymerization [3], monomer-binding proteins stabilize substantial pools of unpolymerized actin. Historically, this G-actin store was believed to be relatively uniform throughout the cytoplasm. Over recent years, however, the concept of functionally distinct G-actin pools has gained attention, largely shaped by monomer-binding proteins such as profilin and thymosin β4 (Tβ4) [4]. Tβ4 sterically blocks other protein binding sites, forming a non-polymerization competent pool of actin. Profilin still allows for controlled actin polymerization and thus provides a polymerization-competent actin pool upon binding to actin [5].

The presence of actin in the nucleus has been widely recognized over the last two decades, and numerous functions of actin in the cell nucleus have been described, including DNA damage repair and replication, chromatin remodeling, and regulation of gene expression [6–8]. As in the cytoplasm, actin exists in both polymerized and unpolymerized forms in the nucleus to perform these diverse functions [9]. Nuclear actin dynamics are controlled by nuclear ABPs [10, 11] and tight regulation of nuclear actin levels [12]. With a molecular mass of 43 kDa and a compact, globular shape, actin is small enough to pass through the hydrophobic nuclear pore by passive diffusion. Nevertheless, active transport pathways exist, underscoring the need to precisely control nuclear actin. Actin export occurs in a complex with exportin 6 and profilin [13]. Nuclear import of actin has been linked to cofilin and importin 9 [14]. This long-standing model has been recently challenged by biochemical evidence suggesting that importin 9 may respond to the availability of free cytoplasmic G-actin and that cofilin may not be strictly required as an import factor [15]. It will be exciting to see how these emerging concepts refine our understanding of nuclear actin homeostasis.

## What this review covers …

To investigate actin’s functions in the cytoplasm and nucleus, a broad toolbox exists to visualize F-actin in live and fixed cells. In contrast, methods for detecting and studying cellular pools of unpolymerized (“G-”) actin are less mature [16]. In this review, we first give a brief overview of common methods for actin visualization to set the context and highlight key caveats. We then revisit the history and molecular basis of the DNase I–actin interaction and discuss fluorescently labeled DNase I as a probe for localizing unpolymerized actin. Finally, we summarize practical considerations for interpreting DNase I staining, with an emphasis on protocol-dependent variability.

## Actin visualization in cytoplasm and nucleus

### Visualization of polymeric actin in the cytoplasm

The tools for visualizing F-actin have been reviewed in detail elsewhere, including their respective advantages and limitations [16, 17]. Here, we therefore only summarize key approaches (Table 1) and highlight caveats that are particularly relevant when F-actin staining is combined with DNase I–based detection of unpolymerized actin.

**Table 1:**
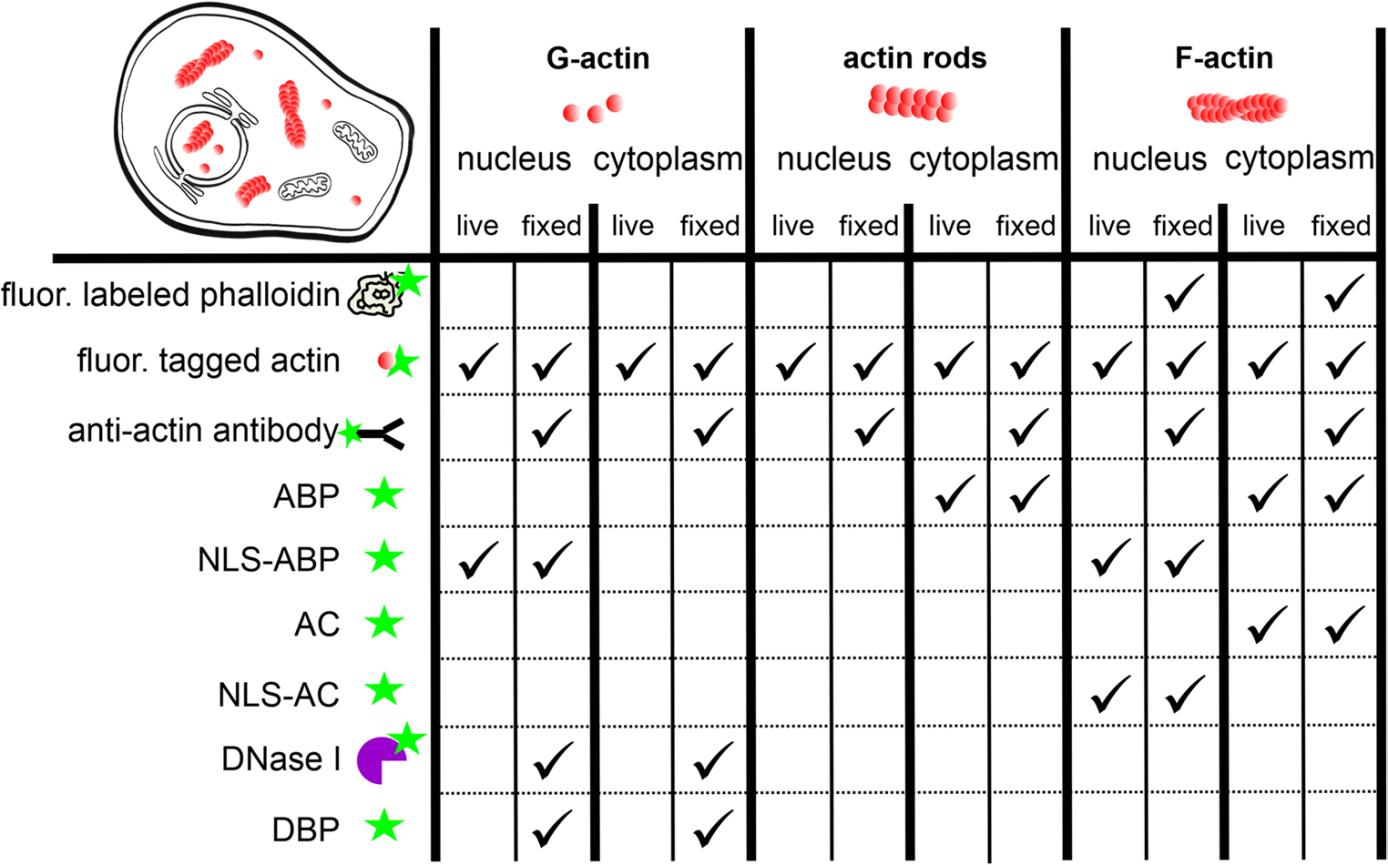
Overview of actin labeling methods. Depending on the antibody used, anti-actin antibodies will label different forms of actin. Many methods originally developed for live-cell imaging can also be applied to fixed cells. Please refer to the main text for more information

### Phalloidin staining

Phalloidin conjugates are the long-standing gold standard for visualizing cytoplasmic actin in fixed cells and tissues. Phalloidin, a toxin derived from the mushroom *Amanita phalloides*, binds specifically to F-actin by inserting into a pocket formed by three adjacent actin subunits within the actin filament [18]. Beyond serving as a stain, phalloidin is a potent F-actin stabilizer that reduces subunit dissociation and thereby inhibits filament depolymerization, a process that is particularly evident at filament ends. Fluorescent phalloidin conjugates are generally not cell-permeant and are therefore primarily used on fixed and permeabilized specimens. Importantly, fixation chemistry strongly impacts staining quality; methanol-based fixatives can disrupt actin and compromise phalloidin labeling, whereas optimized aldehyde-based protocols are commonly preferred. Importantly, however, phalloidin does not label all polymerized actin structures. For example, cofilin-decorated actin rods are not detected by phalloidin staining [19, 20]. These rods have been observed in both the cytoplasm and the nucleus and are associated with cellular responses to various stressors, including heat shock [21–23], oxidative stress [24, 25], and ATP depletion [20, 26]. Paradoxically, although cofilin is generally considered an actin filament-severing protein, it can stabilize filaments under the aforementioned cellular stress conditions. This changes the actin filament twist from right-handed to left-handed, a conformational change that disables phalloidin from binding to actin [19]. Historically, actin rods were visualized in vivo using a non-commercial actin-specific antibody [21, 27, 28]. To our knowledge, many commercially available antibodies do not recognize cofilin-decorated filaments. Rod labeling is therefore often achieved using antibodies directed against cofilin or by expressing genetically encoded reporters [29]. More generally, different anti-actin antibodies can preferentially recognize distinct actin states. A notable example is the actin lower dimer (LD), an unusual oligomeric actin conformation involved in filament junctions, that can be specifically detected only by a particular anti-actin serum [30–32]. Finally, fixation and extraction conditions can strongly affect epitope accessibility and thus influence antibody-based actin labeling - an important general consideration for immunostaining approaches.

**Table:** 

**Information Box 1. Practical considerations for phalloidin staining of F-actin in fixed specimens**
**Interpretation**: Fluorescent phalloidin conjugates are commonly used to visualize F-actin in fixed samples, but the lack of signal does not always mean absence of polymerized actin, as some filament states are poorly labeled. **Fixation as a key determinant**: Actin architecture and labeling quality depend heavily on fixation chemistry and conditions. Use a fixation method that preserves filament integrity and keep fixation time, temperature, and buffer composition consistent across all experiments. **Permeabilization and extraction**: Because fluorescent phalloidin conjugates typically do not penetrate cells, staining is performed after permeabilization. Use the mildest effective permeabilization to avoid disrupting fine actin networks. **Staining conditions**: Phalloidin concentration and incubation time should be optimized empirically to maximize filament contrast while minimizing diffuse background. Apply identical staining and wash conditions across all samples intended for comparison. **Sample handling and washing**: Gentle handling and washing reduce non-specific background and prevent mechanical disruption of the actin cytoskeleton or cell detachment from the substrate. **Controls and imaging comparability**: Include a no-phalloidin control to assess autofluorescence and bleed-through. For comparison, fix, stain, and image using the same methods and settings (illumination, exposure, laser power, detector gain) with consistent display scaling. **Photobleaching**: Minimize light exposure during specimen search and focusing, and image stained samples promptly to reduce bleaching-related bias in qualitative and quantitative comparisons.

The development of F-actin probes, such as LifeAct [33], F-tractin [34], SiR-actin [35], utrophin-based probes [36], and small intracellular anti-actin camelit antibodies, so-called actin-chromobodies (AC) [37, 38], has enabled visualization of dynamic cytoskeletal rearrangements in living cells. However, these probes are not biologically inert: by binding to actin filaments, they can compete with endogenous actin-binding proteins and, depending on probe properties and expression levels, alter filament organization and turnover [16, 39]. Moreover, different probes can preferentially label distinct filament populations or conformational states, potentially biasing the apparent distribution of “F-actin” in vivo. This is particularly relevant for small-molecule probes such as SiR-actin, which are derived from actin-stabilizing compounds and can therefore influence actin dynamics under certain conditions. Consequently, probe choice and dosing/expression should be carefully optimized, and key observations should ideally be corroborated using an orthogonal actin marker or an independent visualization strategy.

**Table:** 

**Information Box 2. F-actin probes at a glance (formats, typical use, key caveats)**
**Phalloidin conjugates (toxin-based; fixed/permeabilized)** - *Strength:* high-contrast labeling of many filament networks. *Caveat:* stabilizes filaments (can “lock” F-actin), if not fixed before, and staining quality depends strongly on fixation/permeabilization; some specialized filament states can be weakly labeled. **LifeAct (17-aa peptide–FP; live/fixed)** - *Strength: *simple, widely used, good for dynamics. *Caveat:* probe-specific biases and potential perturbation are expression-level dependent; “F-actin” becomes defined by the probe. **ABD-based probes (e.g., UtrCH, F-tractin; ABD–FP; live/fixed)** - *Strength: *robust filament labeling; commonly used alternatives/complements to LifeAct. *Caveat:* different ABDs preferentially report different actin networks and can bias distributions; high probe levels can perturb actin. **Actin chromobodies/actin nanobodies (nanobody–FP; live/fixed)** - *Strength*: genetically encoded binders; can be targeted to compartments/structures. *Caveat*: potential state/epitope bias and perturbation at high expression—best interpreted with orthogonal validation. **SiR-actin and related jasplakinolide-derived fluorogenic probes (small molecule; live; super resolution-compatible)** - *Strength: *no transfection; far-red fluorogenic imaging (incl. advanced microscopy). *Cavea*t*: *jasplakinolide-class ligands can stabilize/alter actin dynamics depending on dose/exposure. **Fluorescent actin (actin–FP fusions; live) **- *Strength*: can report incorporation/turnover in some contexts. *Caveat*: not F-actin-specific (labels monomeric + polymeric pools) and can change actin stoichiometry; interpret as a complementary, not primary, F-actin marker.

### Visualization of polymeric actin in the nucleus

Because nuclear F-actin is often low in abundance, highly dynamic, and frequently stimulus-dependent, it can be challenging to detect by phalloidin staining [40]. In addition, some nuclear filament states may be poorly accessible to phalloidin [41]. To visualize nuclear actin in living cells, several reporters have therefore been developed that target established F-actin probes to the nucleus by fusing them to a nuclear localization sequence (NLS), including NLS-LifeAct [42], Utr230-based nuclear probes (e.g., Utr230-EN), and NLS-tagged actin-binding domains fused to a fluorescent protein [43]. One of the most widely used tools following the same principle is an NLS-tagged actin chromobody (nAC) [44]. nAC-based imaging has substantially advanced the mechanistic understanding of nuclear actin function, including roles in chromatin organization [45], transcription [46, 47], and DNA replication [48]. However, it has become clear that nuclear F-actin probes can differ in what they preferentially report and can also perturb nuclear actin dynamics; notably LifeAct and Utr230 have been shown to interfere with nuclear actin behavior and can induce distinct nuclear actin assemblies [49]. Consistent with this, expression of nACs has been reported to promote the formation of nuclear actin fibrils in U2OS cells [50], underscoring the need for careful control of probe levels and, ideally, orthogonal validation with an independent marker or approach.

### Visualization of G-actin in the nucleus

Unlike with F-actin, substantially fewer approaches are available for visualizing G-actin. In living cells, direct imaging of monomeric actin remains challenging; however, canonical G-actin-sensing mechanisms such as the RPEL-based regulation of MRTF/MAL underscore that cells actively monitor monomeric actin pools and have inspired reporter strategies [43]. A probe that specifically recognizes monomeric nuclear actin in live cells suggests monomeric actin is a component of nuclear speckles [43]; to our knowledge, this probe has been used only by the laboratory that developed it. In addition, a limited set of conformation-sensitive antibodies has been described that preferentially recognize non-filamentous actin states (e.g., 2G2 and 1C7) [51–53]. Pan-actin antibodies that bind both globular and filamentous actin conformations, such as AC-15 [54] and C4 [55], have been used to visualize actin pools not readily detected by phalloidin, including nuclear actin structures [52, 56, 57]; for all antibody-based approaches, it should be recognized that fixation/extraction conditions and epitope accessibility can strongly influence the apparent localization pattern.

Vitamin D–binding protein (DBP; Gc-globulin) forms a 1:1 complex with actin [58]. Fluorescently labeled DBP has therefore been used as an affinity probe to visualize actin monomers in fixed cells. Cao, Fishkind, and Wang first introduced DBP-based G-actin detection in 1993 [53]. Although this approach has remained relatively niche, it has reappeared in several subsequent studies [59, 60]. In contrast, fluorescent DNase I conjugates are used far more frequently to stain unpolymerized actin; the DNase I–actin interaction and key considerations for interpreting DNase I staining are discussed below.

**Table:** 

**Information Box 3. Probes to visualize nuclear G-actin (formats, typical use, key caveats)**
**Monomer-preferring nuclear probe/RPEL/MRTF-based (live; indirect)** — *Reports:* probe-defined nuclear “unpolymerized actin” enrichment (incl. speckle-associated patterns), reflecting RPEL–G-actin binding. *Strength*: dynamic readout in living cells.* Caveat:* indirect, G-actin; probe can bias/perturb local equilibria **Fluorescent DNase I (fixed; protein probe) **— *Reports:* “unpolymerized actin” staining in nucleus/cytoplasm. *Strength:* widely used, strong precedent. *Caveat:* monomer specificity not absolute; highly protocol dependent (fixation/extraction/ions) **DBP/Gc-globulin conjugates (fixed; protein probe)** — *Reports:* G-actin–binding signal used as an alternative to DNase I. *Strength:* often discussed as more G-actin–selective. *Caveat:* prep-dependent; competes with profilin and gelsolin for G-actin binding

## DNase I in physiological processes and its applications in research

### Physiological functions of DNase I

DNase I is an endonuclease that was first described in 1905 as an enzyme found in the bovine pancreatic secret [61]. It is a member of the DNase I gene family, which comprises a total of four family members [62]. DNase I nonspecifically cleaves double-stranded DNA, generating 3´-hydroxyl and 5´-phosphate ends. The enzyme’s optimal pH for activity is approximately 7.5, and it requires micromolar concentrations of divalent cations (Ca^2+^, Mg^2+^, or Mn^2+^) [63]. Consequently, enzyme activity can be effectively inhibited by ion chelators, such as ethylene glycol tetraacetic acid (EGTA) or ethylene diamine tetraacetic acid (EDTA).

Early work described DNase I as a secreted protein produced predominantly by organs of the digestive system. Subsequent studies, however, demonstrated that DNase I is broadly expressed in mammalian tissues and is present in organs and body fluids beyond the gastrointestinal tract [64, 65]. This suggested that the physiological function of DNase I extends beyond digestion. Indeed, DNase I has been implicated in DNA clearance during apoptosis [66, 67] and necrosis [68]. Furthermore, a decrease in DNase I level has been described in association with antinuclear autoimmune diseases, such as systemic lupus erythematosus [69, 70]. Mechanistically, it is thought to remove extracellular DNA (ecDNA) derived from soluble or deposited autoantigenic nucleoprotein complexes at sites of high cell turnover, such as the hematopoietic system, thereby limiting immune stimulation and inflammation [71]. Importantly, DNase I activity can be potently inhibited by actin released from damaged cells; in the circulation, this is counterbalanced by the extracellular actin-scavenging system (notably gelsolin and vitamin D–binding protein/DBP), which depolymerizes F-actin and sequesters G-actin. DNase I also contributes to the degradation and clearance of neutrophil extracellular traps (NETs), which are composed of DNA and antimicrobial proteins and are released by neutrophils in a specialized, pathogen-trapping form of cell death called NETosis [72–74]. DNase I clears circulating NETs [75] and thus balances the immune response since excessive or dysregulated NET formation can contribute e.g., to sepsis and sepsis-induced Acute Respiratory Distress Syndrome. In this context, it is also important to consider DNase1L3 as a major secreted serum DNase that degrades antigenic DNA forms (including protein/lipid-associated DNA) and whose deficiency is linked to lupus-like autoimmunity, a condition for which DNase I cannot compensate.

### DNase I treatment as a therapeutic approach

Therapeutic use of DNase I has been explored in multiple animal studies as an approach to target diseases involving circulating ecDNA and NETs. Examples include alcohol-related liver disease [76], acute ischemic stroke [77], traumatic brain injury [78], and tumor-induced vessel impairment [79]. In clinical practice, recombinant human DNase I, marketed as Dornase Alfa (Pulmozyme), has been used for over two decades to treat patients with cystic fibrosis [80]. Delivered as an aerosol, it reduces the viscosity and volume of airway mucus by degrading extracellular DNA in the sputum, and clinical evidence supports improvements in lung function and a reduction in pulmonary exacerbations. Beyond cystic fibrosis, DNase (Dornase Alfa) combined with intrapleural tissue plasminogen activator (tPA) has demonstrated clinical benefit in pleural infection/empyema by improving pleural fluid drainage compared with single agents or placebo. Clinical trials are also underway to evaluate DNase I therapy for reducing excessive NETs formation in patients with sepsis (NCT05453695).

### DNase I-actin – interaction

**Fig. 1:**
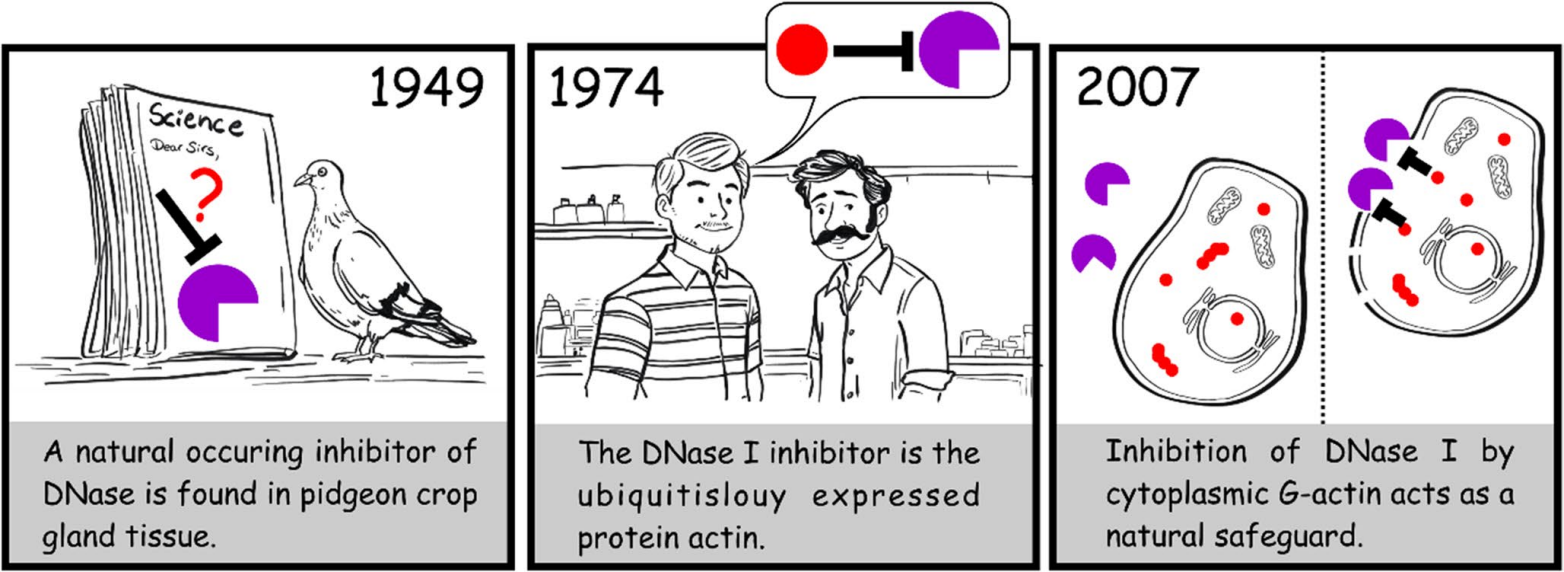
Discovery of actin as a naturally occurring inhibitor of DNase I

In 1949, a naturally occurring inhibitor of DNase I that is found in all tissues was described [81] (Fig. 1). After three decades, this inhibitor was identified as actin [82]. G-actin binds DNase I with a high affinity (5 × 10^8^ M^− 1^) [83], leading to the formation of stable 1:1 complexes. The affinity of DNase I to F-actin is approximately four orders of magnitude lower (1.2 × 10^4^ M^− 1^). Nevertheless, DNase I can still interact with actin filaments in a biologically meaningful manner, in particular by binding at the pointed (-) end. In the actin–DNase I complex, the activities of both proteins are inhibited: actin is prevented from polymerizing, and DNase I nuclease activity is strongly suppressed. Structural studies of the actin–DNase I complex have provided a key framework for understanding this interaction at the molecular level, including the interface involving the actin DNase I–binding loop (D-loop). Beyond DNA degradation, DNase I has also been shown to promote actin filament disassembly. Mechanistically, DNase I can sequester actin monomers, thus hindering actin filament formation and facilitating depolymerization [83]. DNase I also interacts with the pointed end of actin filaments, thereby increasing the rate of filament depolymerization [84–86]. The physiological significance of the DNase I–actin interaction is not yet fully understood. Notably, extracellular actin released from damaged cells can inhibit DNase I, but in circulation, this effect is balanced by the extracellular actin-scavenging system—which includes gelsolin and vitamin D–binding protein (DBP)—that depolymerizes F-actin and sequesters G-actin. Since DNase I has been reported to enter necrotic cells and cells with transiently leaky plasma or endoplasmic membranes, it has been proposed that inhibition of DNase I by cytoplasmic G-actin may serve as a safeguard against premature DNase I activity inside cells [87].

### DNase I fluorescence staining in actin research

In research settings, DNase I is used in a range of applications, including DNase I footprinting/DNase- seq to map regulatory protein occupancy on the genome [88] and for routine removal of DNA contamination from RNA preparations. The high-affinity 1:1 complex formation between actin and DNase I has also been instrumental in structural and mechanistic studies of actin, and it underpins classical DNase I-inhibition-based biochemical assays used to quantify unpolymerized (G-)actin pools [86, 89, 90].

Because direct tracking of G-actin in cells is challenging and DNase I binds actin with very high affinity, fluorescent DNase I has become an established approach for detecting and quantifying unpolymerized actin in cells. The use of rhodamine-labeled DNase I was first described in 1978 as a method for visualizing both actin structures in cells [91]. It was subsequently shown that, in combination with formaldehyde fixation and controlled Triton X permeabilization, DNase I can be used to detect spatially distinct G-actin signals [92–94]. This enabled simultaneous localization and quantification of G- and F-actin using labeled DNase I and phalloidin, respectively. Using this approach, spatial variations in the G-/-F-actin ratio have been reported in cultured cells [94], in plant pollen and pollen tubes [95]. Furthermore, labeled DNase I has been employed to assess how various compounds affect the cellular actin polymerization state [96]. Because DNase I is an endonuclease whose activity depends on divalent cations and can be inhibited by chelators, staining protocols typically avoid conditions that would promote nuclease activity during incubation to minimize DNA-related background.

In recent years, renewed interest in nuclear actin and ABPs has driven a rediscovery of DNase I staining as a tool for monitoring “unpolymerized” actin [97–100]. For example, DNase I staining was used to analyze nuclear actin in Drosophila ovary samples, and supported the existence of multiple pools of nuclear actin that may contribute to different nuclear functions [56]. In addition, changes in nuclear G-actin levels have been linked to the regulation of myocardin-related transcription factor A (MRTF-A): mechanistically, MRTF-A contains RPEL motifs that bind G-actin, and decreases in available G-actin promote MRTF-A nuclear accumulation and SRF-dependent transcriptional activity [97, 98].

### Considerations of DNase I – actin fluorescence staining

In cells, as described above, most G-actin is present in complexes with ABPs. Given the robust DNase I staining typically observed, it is likely that DNase I reports not only “free” G-actin but also ABP-bound pools, depending on whether the respective binding sites overlap and on solution conditions that may modulate steric competition. The binding sites of profilin and gelsolin do not overlap with the DNase I-binding site on actin [101], consistent with DNase I being able to detect profilin-actin and gelsolin-actin complexes. G-actin bound to members of the ADF/cofilin family is also likely recognized, supported by the purification of actin-cofilin complexes via DNase I affinity chromatography [102]. Similarly, because the Tβ4 binding site on actin differs from the DNase binding site, ternary complexes of DNase I with Tβ4:actin can form under crosslinking conditions [103].

Actin-related proteins (Arps) are a family of proteins that share a high degree of sequence homology with actin. Despite this homology it should be noted that there is no experimental evidence that DNase I binds to Arps. Especially nuclear Arps exhibit truncated or divergent DNase I binding loops [104, 105] which makes DNase I binding unlikely.

Collectively, these findings suggest that DNase I staining captures a broad pool of unpolymerized actin. Importantly, however, DNase I is not strictly monomer-exclusive: beyond its high-affinity binding to G-actin, DNase I can also interact with actin filaments, in particular at filament pointed (-) ends, which should be considered when interpreting staining patterns – especially in regions enriched in filament ends or remodeled filaments.

**Fig. 2:**
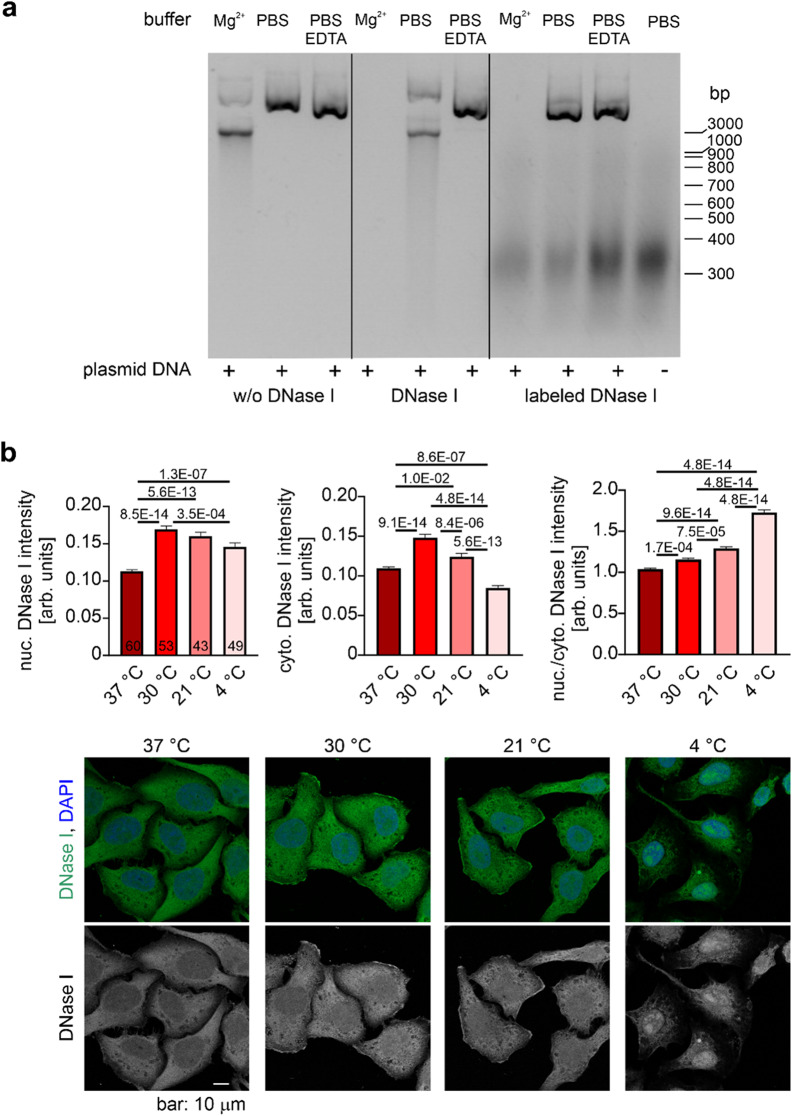
DNase I activity and staining patterns under different experimental conditions. **A**) Agarose gel electrophoresis of plasmid DNA after treatment with DNase I. Plasmid DNA was incubated without DNase I, with commercially available DNase I, and with labeled DNase I under different buffer conditions. Enzyme activity was observed in Mg^2+^-containing buffer (Mg^2+^) but not in PBS or PBS with EDTA. **B**) HeLa cells were washed with PBS and fixed in 4% PFA in PBS at the indicated temperatures. Cells were extracted using 0.2% Triton X in PBS and stained with Alexa Fluor 488–conjugated DNase I (1:500 in PBS, Invitrogen) and DAPI. A LSM800 (Zeiss) confocal laser scanning microscope equipped with a 40 × 1.3 oil immersion objective was used for microscopic analysis. The confocal pinhole was set to 1 Airy unit, and images were taken using a multi-track acquisition mode in the LSM Zen Blue software using the same settings for all images taken. Images were analyzed using the CellProfiler software. DAPI staining was used to identify the nuclear area. Cytoplasmic area was identified by DNase I staining omitting the nuclear area. The numbers in the bars indicate the number of cells analyzed. Data are presented as mean ± SEM with statistical analysis performed using one-way ANOVA followed by Tukey post-hoc test

Because DNase I is frequently used to monitor nuclear G-actin levels, questions about potential residual nuclease activity of fluorescent DNase I conjugates are important. For commercially available fluorescently labeled DNase I, information on enzymatic activity is typically not publicly documented and is not always provided upon request. In our experiments (Fig. 2a), the purchased labeled protein retained detectable nuclease activity under permissive buffer conditions. However, when diluted in PBS, to mimic common staining conditions, no DNase I activity is observed, consistent with the lack of Ca^2+^ and Mg^2+^ in PBS and the known cation requirement for DNase I activity. In addition, in cellular contexts, any residual DNase I activity is expected to be strongly dampened by the high-affinity inhibitory interaction with actin, further reducing the likelihood of DNA digestion under typical staining conditions.

Whether enzymatically inactive DNase I can still bind nuclear DNA in fixed cells remains unresolved. One study proposed that, beyond supporting catalysis, bound Ca²⁺ and Mg²⁺ can also tune the electrostatic “fit” of DNase I for DNA and thereby influence DNase I – DNA binding [106]. More generally, mechanistic work suggests that multiple DNase I ion-binding sites can assist both DNA binding and hydrolysis, making ionic conditions a plausible determinant of nuclear DNA-associated background in fixed samples [106]. The authors noted that crystallographic DNA/DNase I complexes [107] appear to conflict with this view, potentially due to ions that are not detected under certain crystallographic conditions. Observations in fixed samples also argue against strong chromatin binding in cells: in Drosophila follicle cells, nuclear DNase I staining was reported not to overlap with chromatin [56], and DNase I staining was similarly described as spatially separated from DNA in migrating cells [94]. In practice, this underscores the value of explicitly assessing DNase I spatial overlap with a DNA stain (e.g., DAPI) within the same imaging/analysis pipeline when interpreting nuclear DNase I patterns.

A very practical issue in fluorescent DNase I staining is the pronounced sensitivity of the readout to sample preparation, making protocol choice – and especially fixation conditions – critical experimental variables. Common workflows include paraformaldehyde (PFA) fixation followed by acetone permeabilization or, alternatively, PFA fixation followed by detergent permeabilization (e.g., Triton X); the latter has been reported to yield a more specific readout of G-actin. However, fixation temperatures are rarely specified in published protocols and, when reported, vary widely across studies, ranging from 4 °C to 37 °C. Importantly, our data show that fixation temperature alone is sufficient to shift DNase I staining intensity in both the nucleus and cytoplasm (Fig. 2b). We therefore recommend treating fixation temperature as a controlled and explicitly reported parameter – together with fixative composition, fixation time, and permeabilization conditions – and keeping it constant within and across experimental batches to ensure reproducible and interpretable DNase I staining outcomes.

**Table:** 

**Information Box 4. Key points for DNase I staining of nuclear “G-actin”**
• DNase I staining provides a widely used, high-affinity readout of a pool of “unpolymerized” actin in fixed cells. • DNase I is **not perfectly monomer-exclusive**: it can bind actin filaments in vitro and can interact with filament ends; interpretation should therefore be cautious, especially in nuclei. • The apparent specificity and subcellular distribution of DNase I signal are **highly protocol-dependent**, with fixation and permeabilization/extraction conditions being major determinants. • Because DNase I is a nuclease whose activity depends on divalent cations, staining conditions should minimize any residual nuclease activity and DNA-related background. • Combining DNase I with orthogonal actin markers supports the concept of **multiple**,** reagent-distinct nuclear actin pools**. • Robust conclusions require **appropriate controls and orthogonal validation**, rather than reliance on a single probe **Recommended baseline protocol** 1. Wash: 3 x PBS (37 °C), at 37 °C 2. Fix: 4% paraformaldehyde in PBS (37 °C), 20 min at 37 °C 3. Wash: 3 x with PBS 4. Permeabilize: 0.2% Triton X-100 in PBS for 5 min 5. Wash: 3 x with PBS 6. Stain: Conjugated DNase I in PBS (determine optimal dilution in advance). Stain for 30 min. 7. Wash: 3 x with PBS 8. Counterstains (optional): Phalloidin (F-actin), DAPI (DNA) 9. Wash 3 x with PBS; mount - Never include **Mg**^**2+**^/**Ca**^**2+**^ in any incubation with labeled DNase I - Consider adding **EDTA/EGTA** in stain/washes - record **temperature** during fixation; ideally wash and fix at 37 °C

Although its physiological function remains incompletely understood, DNase I has been established as a valuable therapeutic agent and research tool since its discovery in 1905. Labeled DNase I is one of the few ways to preferentially stain G-actin in cells. There are several points to consider when using DNase I labeling, including that DNase I also binds to the pointed ends of actin filaments and that the staining outcome depends on the fixation temperature. Although many labeling methods have been developed to analyze the actin cytoskeleton in different settings, the toolbox for investigating monomeric actin remains somewhat limited. Therefore, DNase I staining will continue to be used as a simple and quick method.

**Table:** 

**Information Box 5. Quantification guidance**
**Define the readout(s) up front**: What exactly is being quantified from DNase I (e.g., “DNase I fluorescence as an unpolymerized-actin signal”), and what is being quantified from the comparator (phalloidin F-actin, pan-actin, etc.). **Recommended primary metrics**: - **Nucleus-to-cytoplasm ratio (N/C)** of DNase I intensity (robust across imaging sessions). - **Mean nuclear intensity** - **Fraction of DNase I signal in nucleus** (integrated intensity nucleus/whole cell). If relevant: **speckle-associated enrichment** (e.g., co-localization with a speckle marker; enrichment over nucleoplasm). **Segmentation strategy**: How to define **nucleus**,** cytoplasm**, and optional compartments (nucleoli excluded, perinuclear ring excluded, etc.). Use DAPI/Hoechst for nuclei; define cytoplasm via cell mask or a cytoplasmic stain. State whether you exclude mitotic cells, multinucleated cells, or edge cells. **Normalization**: Recommend normalization options: - Normalize to exposure time/laser power; keep settings fixed. - Use internal reference samples per batch. - Avoid normalizing DNase I by total actin unless the biological question demands it (can confound). **Acquisition comparability**: Same microscope settings across conditions: objective, pinhole (confocal), laser power, gain, offset, z-step, exposure, bit depth. Avoid saturation; verify linearity. Keep display settings separate from quantification. **2D vs. 3D (especially for nuclei)**: State whether you quantify from a **single plane**,** max projection**, or **3D stack**. For nuclear signal, a 3D or standardized midplane is often more reproducible. Be explicit, because projections can inflate the nuclear signal. **Batch effects & replication design**: Biological replicates (independent cultures), technical replicates, number of cells per replicate, and how fields are sampled (random fields vs. “nice-looking cells”). Include a rule like: “sample across ≥ 3 independent experiments”. **Outlier handling and inclusion criteria**: Predefine criteria (e.g., exclude damaged nuclei, apoptotic/pyknotic, over-permeabilized cells, cells with clear extraction artifacts). Avoid post hoc removal without rules.

## Outstanding questions, outlook

Despite its long history, several questions remain central to interpreting DNase I staining in the context of nuclear actin. First, what fraction of the nuclear DNase I signal reflects truly monomeric actin versus alternative unpolymerized or short-polymer states, and how strongly is this influenced by sample preparation? Second, how do ionic conditions and potential DNA interactions contribute to nuclear background, and can standardized staining buffers minimize variability across laboratories? Third, how do DNase I–defined nuclear actin pools relate to pools defined by conformation-sensitive antibodies and by live-cell nuclear actin reporters—do these reagents report overlapping species or fundamentally distinct actin states? Addressing these points will be important for further establishing DNase I staining as a robust, quantitatively interpretable tool for studying nuclear actin biology.

## Supplementary Information

Below is the link to the electronic supplementary material.

Supplementary Material 1

## Data Availability

The data are available upon request.
