# Supplementary figures and images for: DNase I as a probe for unpolymerized actin: revisiting a classic tool for nuclear actin research
Source: Pflugers Arch. 2026 Mar 26;478(4):32. doi: 10.1007/s00424-026-03158-z (PMC13018090; doi:10.1007/s00424-026-03158-z)

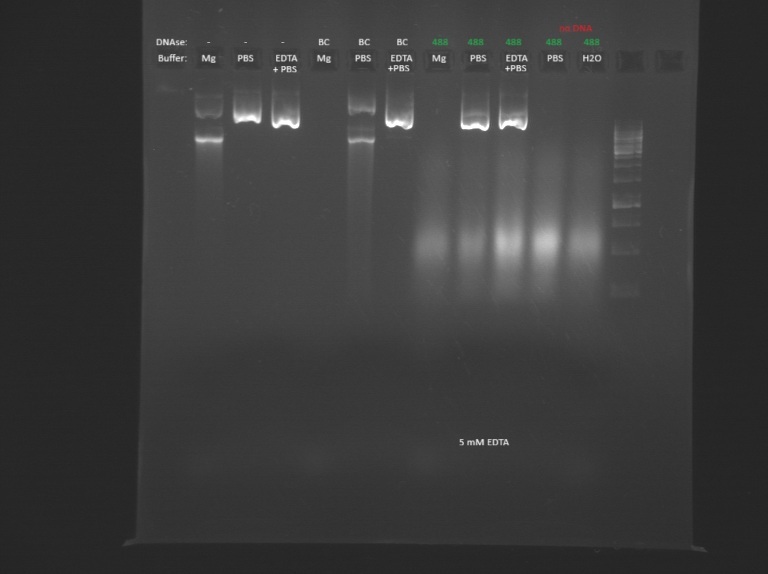

Supplement: Supplementary file 1 — Supplementary Material 1 [file 424_2026_3158_MOESM1_ESM.jpg]
